# Research progress and management strategies of fungal diseases in *Camellia oleifera*

**DOI:** 10.3389/fmicb.2023.1215024

**Published:** 2023-11-23

**Authors:** Xingzhou Chen, Yuan He, Zhikai Wang, Anqi Niu, Yi Xue, Diao Zhou, Guoying Zhou, Junang Liu

**Affiliations:** ^1^Key Laboratory of Cultivation and Protection for Non-Wood Forest Trees, Central South University of Forestry and Technology, Changsha, China; ^2^Key Laboratory of National Forestry and Grassland Administration on Control of Artificial Forest Diseases and Pests in South China, Central South University of Forestry and Technology, Changsha, China; ^3^Hunan Provincial Key Laboratory for Control of Forest Diseases and Pests, Central South University of Forestry and Technology, Changsha, China

**Keywords:** *Camellia oleifera*, fungal pathogens, plant diseases, management strategies, geographical distribution

## Abstract

*Camellia oleifera* Abel, a woody oil plant, that is endemic to China. Tea oil, also referred to as “oriental olive oil,” is a superior quality plant-based cooking oil. The production of tea oil accounts for 8% of the total edible vegetable oil production in the country. Since 2022, the annual output value of *C. oleifera* industry has exceeded 100 billion yuan, making it one of the major economic contributors to China’s rural revitalization development strategy. In recent years, demand and production have grown in parallel. However, this has led to an increase in the incidence levels of pest and diseases. Pests and diseases significantly reduce the quality and yield of *C. oleifera*. *C. oleifera* diseases are mainly caused by pathogenic fungi. *C. oleifera* anthracnose, soft rot, leaf spot, coal stain, leaf gall disease, and root rot are the most important fungal diseases affecting the *C. oleifera* industry. However, the same disease may be caused by different pathogenic fungi. *C. oleifera* can be found in half of China and is found in several climatic zones. The geographical distribution of woody plant diseases is consistent with the distribution of the tree species and the ecology of the range, which also results in a highly complex distribution of fungal diseases of *C. oleifera*. The management of fungal diseases in *C. oleifera* is extremely challenging due to the variety of pathogenic fungal species, multiple routes of transmission, the lack of resistant plants, and the environmental safety of chemical measures. The optimal strategy for addressing fungal diseases in *C. oleifera* is to develop and apply an integrated disease management plan. This review provides a brief overview of the pathogenic species, pathogenesis, pathogenesis, geographical distribution, current management strategies, and potentially new methods of *C. oleifera* fungal diseases, to provide direction for the development of comprehensive management measures for *C. oleifera* fungal diseases in the future.

## 1 Introduction

*Camellia oleifera* Abel has been cultivated for more than 2,000 years in China, and the domestic cultivation area is the largest in the world (He and He, 2002). In addition, it is minor planting in Japan, South Korea, India, Southeast Asia, and certain Western countries (Aukkanimart et al., 2013; Suealek et al., 2018; He et al., 2021; Teixeira and Sousa, 2021). *C. oleifera* is a leading industry in China’s rural areas, promoting it enriching the people, and serving as a livelihood industry to combat poverty. Since 2022, the national *C. oleifera* planting area reached 467 million hectares, with a total output of tea oil of 1 million tons, and a total output value of the industry exceeding 1,160 billion yuan (The Central People’s Government of the People’s Republic of China, 2020; National Forestry and Grassland Administration, 2023). Tea oil boasts high nutritional value and an array of healthcare benefits (Hong et al., 2019; Du et al., 2020), including aging inhibiting, assistance to pregnant women during lactation and postpartum recovery (Zhang et al., 2019), antioxidant (Zhong et al., 2019), anti-cancer properties (Zong et al., 2016; Zheng et al., 2019), among others. In recent years, the Chinese government has strongly supported the expansion of the *C. oleifera* plantation area to foster the development of the advantageous *C. oleifera* industry (National Forestry and Grassland Administration, 2021; Central People’s Government of the People’s Republic of China, 2022). However, as the cultivation area of *C. oleifera* continues to increase, the occurrence of diverse diseases is increasing daily. During previous investigations, it was discovered that plant pathogenic fungi were the primary causes of diseases in *C. oleifera.* This causes the flower and fruit drop rates of *C. oleifera* to increase up to 70–80% in certain regions. The traditional diagnosis of diseases in *C. oleifera* often depends on the practitioner’s experience, with symptoms serving as the sole basis for deducing the disease type. However, the symptoms vary due to different pathogenic fungi in various environments, different stages of infection, and different plant tissues, resulting in confusion in the classification of *C. oleifera* disease. Researchers were not able to develop the fact that there is a wide range of pathogenic fungi causing *C. oleifera* disease and that the same disease can be caused by multiple pathogenic fungi until the advent of molecular tools (Zhu and He, 2023). The natural geographic distribution of woody plant diseases is closely linked to the distribution of tree species, pest biota, and ecological environment. *C. oleifera* is widely grown and can survive in complex environments, therefore, analyzing the distribution of fungal diseases in *C. oleifera* will aid in the implementation of integrated disease management strategies. Targeted and precise prevention and management of plant diseases is a hot topic of discussion in the academic community, which cannot be achieved without an in-depth investigation into the pathogenic mechanisms of pathogenic fungi. Emerging research findings on the causal fungi of *C. oleifera* fungal diseases are emerging, which providing a strong basis for new integrated management strategies. The majority of reviews pertaining to fungal diseases in *C. oleifera* merely provide a brief summary of the conditions under which they occur and the pathogenic fungal species involved, lacking detailed descriptions of their geographical distribution, pathogenic fungi, and the mechanisms of their pathogenicity. Furthermore, as more and more novel pathogenic fungi are identified, it is crucial to promptly summarize these details. The purpose of this review is to provide summary of the present condition of the primary fungal diseases affecting *C. oleifera* (pathogenic species, geographical distribution, pathogenic mechanisms). Also, discuss potential integrated management strategies for *C. oleifera* disease in the context of existing research.

## 2 Current occurrence of major fungal diseases of *Camellia oleifera*

Common fungal diseases of *C. oleifera* include anthracnose, soft rot, leaf spot, coal stain, leaf gall, and root rot. These diseases spread rapidly and can cause significant harm, often resulting in the widespread death of *C. oleifera* in severe cases. Rarely have white rot, scab disease, and algal spot disease of *C. oleifera* been reported, and systematic studies are lacking. Hence, solely the corresponding pathogenic fungal species are presented in this paper (Table 1).

**TABLE 1:** Statistics on the names of essential fungal diseases and the types of pathogens in *Camellia oleifera.*

Name	Another name	Pathogens
*Camellia oleifera* anthracnose	/	*Colletotrichum fructicola* (Li et al., 2016), *C. gloeosporioides* (Li H. et al., 2017), *C. camalliae* (Wang Y. et al., 2020), *C. siamense* (Li et al., 2015), *C. aenigma* (Wang Y. et al., 2020), *C. karstii* (Jiang and Li, 2018), *C. nymphaeae* (Li and Li, 2020), *Colletotrichum kahawae* (Sun W. et al., 2022), *C. boninense* (Tang et al., 2015), *C. horii* (Sun W. et al., 2022), *C. fioriniae* (Qin et al., 2019a), *Glomerella cingulata* (Li H. et al., 2017)
*Camellia oleifera* soft rot	“Deciduous disease,” “Leaf blight”	*Agaricodochium camelliae* Liu Wei ei Fan (Liu et al., 1981; Song et al., 2021)
*Camellia oleifera* leaf spot	“Red leaf spot” “Gray spot disease,” “Purple spot disease,” “Frogeye leaf spot”	*Phyllosticta theicola* Petch (He, 2016), *Neopestalotiopsis longiappendiculata* (Wang Q. T. et al., 2022), *Cercosporella theae* (Huang and Meng, 1986), *Phyllosticta theaefolia* Hara (Liu, 1964), *Pestalozzia Palmarm* Cooke (Liu, 1964), *Leptosphaeria* sp (Kirk et al., 2008), *Mycosphaerella* sp (Kirk et al., 2008), *Macrophoma* sp (Kirk et al., 2008), *Ascochyta* sp (He, 2016), *Lasiodiplodia theobromae* (Liu et al., 2019), *Epicoccum layuense* (Xie et al., 2022), *Haradamyces foliicola* (Lu et al., 2019), *Neofusicoccum parvum* (Yu et al., 2022), *Botryosphaeria dothidea* (Hao et al., 2022), *Nigrospora sphaerica* (Qin et al., 2021), *Nigrospora chinensis* (Liu et al., 2020), *Pestalotiopsis microspora (Li et al., 2011)*, *N. cubana*, *N. iberica*, *N. camelliae-oleiferae*, *N.* sp.1, *P. camelliae-oleiferae*, *P. hunanensis*, *P. nanjingensis*, *P. naningensis* (Li et al., 2021), *P. cocculin* (Qin et al., 2019b), *Pestalozzia theae* Saw, *Athelia scutellare* (Xie et al., 2022), *Alternaria alternata* (Shi et al., 2015)
*Camellia oleifera* coal stain	“Coal disease”	*Capnodium theae* Hara, *Meliola camelliae* (Catt) Sacc (Liu, 1964; Chinese Academy of Forestry, 1984; Yang, 1996; Zhuang, 2008)
*Camellia oleifera* leaf gall	“Tea blister blight,” “Tea bud disease,” “Tea peach,” “Cha Pao,” “Cha Pian”	*Exobasidium gracile* (Shi-rai) Syd (Lee et al., 2015; Zhang T. T. et al., 2022)
*Camellia oleifera* algal spot	/	*Cephaleuros virescens* Kunze (Su and Zhang, 2009)
*Camellia oleifera* scab disease	/	*Monochaetia* sp (Liu, 1964; Zhao and Qin, 2015)
*Camellia oleifera* white rot	“Ban bianfeng,” “Bai xiu disease,” “Foot rot,” “Plaster tree,” “White dry rot”	*Corticium scutellare* Brek. et Cur (Yuan et al., 1997)
*Camellia oleifera* root rot	“Athelia rolfsii,” “Mycorrhizal disease,” “Mycorrhizal root rot”	*Sclerotium rolfsii* (Liu, 1964), *Athelia rolfsii* (Li et al., 2009), *Fusarium oxysporum* (Zhao et al., 2020), *Fusarium proliferatum* (Li et al., 2008), *Cylindrocarpon destructans* (Zins.) Scholten (Cao et al., 1993)

**FIGURE 1:**
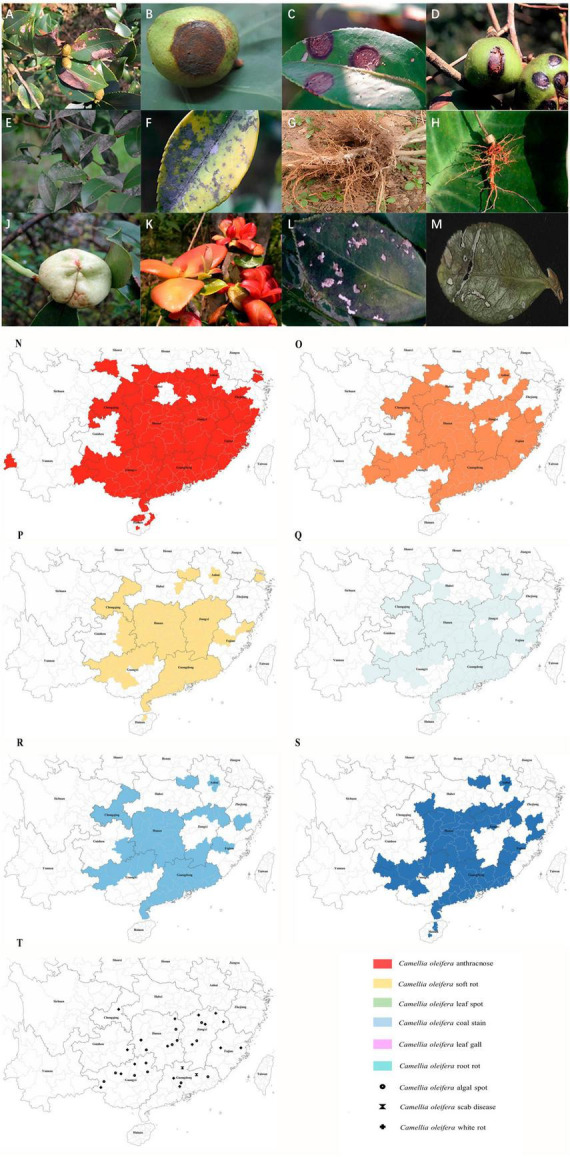
Images showing symptoms of major diseases of *Camellia oleifera* and geographical distribution of the main fungal diseases. **(A)** Anthracnose (leaf), **(B)** Anthracnose (fruit), **(C)** Soft rot (leaf), **(D)** Soft rot (fruit), **(E,F)** Coal stain (leaf), **(G,H)** Root rot, **(J)** Leaf gall (fruit), **(K)** Leaf gall (leaf), **(L)** Leaf spot (caused by *Pestalotiopsis theae*), **(M)** Leaf spot (caused by *Neopestalotiopsis longiappendiculata*), **(N)** Distribution of *C. oleifera* anthracnose, **(O)** Distribution of *C. oleifera* soft rot, **(P)** Distribution of *C. oleifera* leaf spot, **(Q)** Distribution of *C. oleifera* coal stain, **(R)** Distribution of *C. oleifera* leaf gall, **(S)** Distribution of *C. oleifera* root rot. **(T)** Distribution of *C. oleifera* algal spot, white rot and scab disease. Geographical distribution statistical criteria: the disease is mainly controlled or severely damaged in the area. See Supplementary material for reference.

*C. oleifera* anthracnose has been the primary disease. It can cause severe fruit drop, fall buds, branch tip dieback, and even whole plant decline. Due to this disease, *C. oleifera* yields are frequently reduced by 10–30% in all provinces (districts), and by up to 40–80% in heavily affected regions (Zhou, 2023). The fungus of the genus *Colletotrichum* is the primary cause of *C. oleifera* anthracnose, with 12 identified species of *Colletotrichum* (Teleomorph: *Glomerella cingulata*) capable of infecting *C. oleifera* (Table 1). *Colletotrichum fructicola* is the predominant pathogenic fungus that cause anthracnose in *C. oleifera*, which is widely distributed across all *C. oleifera* growing areas in China (Li, 2018). *Colletotrichum* spp typically invades *C. oleifera* tissues in two forms, one by producing infestation pegs that can directly penetrate the cuticle of *C. oleifera*, and the other way is by producing bud tubes from conidia or invading stomata with their mycelium (Li, 2021). *Colletotrichum* spp as a typical semi-living trophic fungus with both living and dead trophic phases. During the live nutrient stage, the structural or functional integrity of the pathogenic fungal infestation is critical in determining it’s to successfully invade plant tissues. However, certain mutants can lose their pathogenicity due to conidia not properly germinating (Liang et al., 2021; Lee et al., 2022). In addition, the formation of the appressorium of plant pathogenic fungi and the strong turgor pressure within it determine the host penetration mediated by compression. Studies have shown that the deletion of autophagy-related proteins CfAtg8 and CfAtg9 leads to a reduction in the melanin layer of the appressorium of *C. fructicola*, accompanied by cytorrhysis and plasmolysis, ultimately affecting the pathogenicity of the pathogenic fungi (Zhang S. et al., 2022). Necrotrophic stage pathogenic fungi are more likely to interfere with plant immunity or induce plant cell death, which consequently fosters further infestation activity. If important PAMPs (es) or DAMPs (Damage associated molecular patterns) are absent, the causative agent cannot suppress the immune responses to ROS, callose, and hormone accumulation in plant tissues, leading to decreased virulence (Balmer et al., 2013; Sun et al., 2016). Anthracnose follows a distinct seasonal pattern, with the average daily temperature beginning to rise from early April, and the minimum dew-point temperature reaching approximately 24°C, moisture condenses on the leaves, and the pathogen begins to infect the young leaf and tip tissues (Figure 1A; Noriega Cantú et al., 2017). The peak was reached in August, and along with the phenology changes in *C. oleifera*, a large number of fruits were infected (Figure 1B). As temperatures and air humidity decrease at the end of October, the disease tends to slow down (Jing et al., 2009; Liu, 2012). The mycelia or conidia of the pathogen overwinter in the tissue of the spot, young fruit, or receptacle, serving as the origin of the initial infestation the following year (Liu, 2012; Wang et al., 2013).

In recent years, the occurrence of *C. oleifera* soft rot has been frequent and severe. In 1981, *Agaricodochium camelliae* Liu Wei ei Fan as the causal fungus responsible for causing *C. oleifera* soft rot (Liu et al., 1981). Later, the morphology of its conidiophores and conidia led to it also being referred to as *Myrothecium camelliae* (Liu Wei ei Fan) P. K. Chi, Wu ex Lin (Qi and Wei, 1983). In 2013, *A. camelliae* was included in the *National List of Hazardous Pests in Forestry* (National Forestry and Grassland Administration, 2013). *A. camelliae* differs from the majority of plant pathogenic fungi in that its invasion is directly achieved by sporodochium rather than conidia. During an infestation, sporodochium grows parallel brush-like mycelial tufts, invading the plant cells through the stomata or penetrating the epidermis. As the invasion process progresses, the sporodochium gradually shrinks (Wei and Qi, 1982). It mainly impacts the leaves and fruits of *C. oleifera*. In severe cases, up to 95% of the plants and 50% of the fruits can be affected by the disease (Figures 1C, D; Jiang, 2007; Wang Y. C. et al., 2020). The principal characteristic of the disease is the development of waterlogged state spots that are yellowish-brown or brownish-yellow, mushroom-like sporodochium. During the late period, the spots are scattered with earthy-yellow mushroom-like sporodochium, and the spots rot, causing the diseased fruit to split (Qi and Wei, 1983; Wei and Ma, 2010). The onset of *C. oleifera* soft rot is affected by the ambient temperature and humidity, with two peak development periods occurring from April to June and October to November each year, when temperatures average between 15–25°C. Temperatures that are too high or too low can hinder the progress of the disease (Lin et al., 1981; Wang Y. C. et al., 2020; Lu, 2021).

*Capnodium theae* Hara and *Meliola camelliae* (Catt) Sacc are recognized as the pathogenic fungi that cause *C. oleifera* coal stain. According to the nutritional mode, some scholars believe that bituminous coal pathogens can be classified into parasitic and saprophytic types (Yamamoto, 1955). As saprophytic type fungi, *Ca. theae* mainly derives nutrients from nectar secreted by insects. Spore germination and shoot tube elongation occur only in the presence of honeydew from insects or secretions of plants. Stimulation of germination and growth is not achieved through ordinary sugar water or nutrient solutions (Yamamoto, 1952). This type of fungus does not cause direct harm to plant tissues, instead, it covers the surface of branches and leaves, influencing photosynthesis and gas exchange, thus causing plant damage indirectly (Reynolds, 1999). Disease outbreaks caused by this pathogenic fungus are usually accompanied by severe insect infestations (Chomnunti et al., 2014; Khalkho et al., 2021). In contrast, *M. camelliae*, as a parasitic type, typically infects the extrafloral nectaries region of plant leaves and stems, drawing nutrients from plant tissues to fuel its growth (Mesquita et al., 2022). Severe cases of *C. oleifera* coal stain have been documented to cause a reduction in yields by 60–70%, which can persist for years to come, ultimately leading to no harvest (Chen, 1980). The key characteristics of *C. oleifera* coal stain are the presence of black sooty spots on branches and leaves during the initial stages, and black mycelium covering the entire leaf in the later stages (Figures 1E, F). The pathogenic fungi have a preference for cool, highly humid conditions, with the highest prevalence of the disease occurring during the months of March to May and September to November (Yu, 2019).

*Camellia oleifera* root rot can be subdivided into two categories (Figures 1G, H). Initially, it was believed that *Sclerotium rolfsii* (Teleomorph: *Athelia rolfsii*) was the causal agent of *C. oleifera* root rot (Table 1). The study discovered that *S. rolfsii* tends to infect the stems of plants close to the soil, but it can also damage various tissues, including shoots, buds, and petioles (Xu et al., 2008; Balamurugan et al., 2022; Thomas and Saravanakumar, 2023). Plant diseases caused by *S. rolfsii* are therefore also referred to as stem rot or blight. *S. rolfsii* can be found in soil or soil surface residues in the form of mycelia and sclerotia, utilizing two infection modes to fully colonize, including invading from damaged plant tissues and directly infecting the plant’s soft tissues (Bhuiyan et al., 2019). The pathogenic fungus produces the virulence factor oxalic acid, which inhibits the host’s defenses and causes stomatal opening, ultimately leading to increases transpiration rates and reduced biomass, resulting in wilting (Guimarães and Stotz, 2004; Kabbage et al., 2015). Infected plants of *S. rolfsii* produce white mycelial mats on the surface of the lesion, which are followed by the formation of globular nuclei either at the infection site or in the surrounding soil (Han et al., 2012; Amaradasa et al., 2020). *C. oleifera* is predominantly a seedling disease, and infection with *S. rolfsii* causes lesions to appear near the ground at the base of the stem or at the root base, which indicates a covering of white mycelium and dark-colored pycnidia. The affected seedlings grow poorly, their stem rots, their leaves turn yellow and wilt, and they can be easily pulled up (Huang and Xiao, 2015). Thus far, there have been no reports indicating that *S. rolfsii* causes harm to the buds, fruits, and other tissues of *C. oleifera*. In recent years, more *Fusarium* spp have been isolated and identified from diseased *C. oleifera* (Table 1). As weak parasites, they specialize in infesting plants that are poorly grown or have a lower resistant. Normally, they are harbored in the soil as saprophytic nutrients or spores that sprout and grow upon stimulated by plant root secretions (Ding et al., 2020). *Fusarium* spp tends to invade from the root tips of the plant, resulting in symptoms like yellowing of the leaves, reduced leaf size, and plant dwarfism. Some pathogenic fungi enter the vascular bundles and secrete pectinase and cellulase enzymes that degrade cells, and block the transport of water and nutrients. As a result, the plants exhibit symptoms of water loss and wilt (Garcia-Maceira et al., 2001). In addition to this, *Fusarium* spp secretes toxins, including monocotyledons and fusaric acid, that aid in the rapid colonization of disease-causing fungi by inhibiting protein synthesis, altering plant cell growth, mitochondrial activity, membrane permeability, or inducing programmed cell death in plant cells (Bouizgarne et al., 2006; Jiao et al., 2013). It’s worth noting that the toxin produced by *Fusarium* spp maintains a high degree of toxicity to humans and animals as well. Accidental ingestion of infected fruits or seeds may pose a life-threatening risk (Qiu et al., 2019). According to survey statistics, the incidence of *C. oleifera* root rot in seedlings is as high as 50%, and in severe cases, it can reach 30% or more in young stands (Zhao et al., 2020). *Sclerotia* spp is maintained at 15–25°C for 27–34 days to initiate germination, and higher soil moisture accelerates this process (Clarkson et al., 2004). Similarly, *Fusarium* spp demonstrated increased pathogenicity at around 25°C, with increased humidity accelerating the development of the disease (Marin et al., 1995; Lui and Kushalappa, 2002). The incidence of both types of root rot peaks during July to August (Li et al., 2008; He, 2016; Ma and Luo, 2019).

*Camellia oleifera* leaf gall drew attention in the 1940s (Yang, 1941). The pathogenic fungus causing the lesions is *Exobasidium gracile* (Shi-rai) Syd. Over 170 species of *Exobasidium* spp are plant pathogens, all of which share the common trait of infecting young, tender plant tissues, leading to the formation of leaf and flower galls (Figures 1J, K; Weille, 1960; Nickerson and Kloet, 1997). Currently only *E. gracile* is found in *C. oleifera* (Dong et al., 2019). After infecting the young flower and leaf buds of *C. oleifera*, the pathogen stimulates tissue proliferation and cell expansion, resulting in the formation of gall bodies (Liu et al., 2012; Huang et al., 2017). Once the flower buds proliferate, they do not produce fruit and seeds, resulting in reduced yields. The proliferation of leaves disrupts the usual process of photosynthesis, consequently leading to reduced yields. Documented that the average incidence of *C. oleifera* leaf swelling is around 39%, with a maximum of 80% or more causing serious damage (Qiu et al., 2011; Jia et al., 2017). Most *Exobasidium* spp produce both basidiospores and conidia, although both types of spores are capable of infection. However, conidia are more resilient, and the causal fungus often attaches itself to the tissue, surviving through winter or summer as the initial. The basidiospore is constrained by its structural specificity and serves primarily for dissemination (Ingram et al., 2019). The basidiospores are often present following the onset of disease, thus challenging the prediction of disease occurrence by detecting spores in the air. *C. oleifera* leaf gall often occurs in early spring, and a warm and humid environment is favorable for the disease. However, when the temperature exceeds 29°C, the growth of the pathogenic fungi is restricted, and the disease occurrence is slowed down (Ingram et al., 2019). Interestingly, *C. oleifera* leaf gall can reduce yields and affect the plant’s health. However, the galls are edible and have a fresh and sweet taste. In addition, it is rich in nutrients and has been found to have hypoglycemic and anticancer effects (Zhu et al., 2007a,b; Huang et al., 2018).

*Camellia oleifera* leaf spot generally refers to a disease that cause leaf damage and form “leaf spots,” including anthracnose and soft rot disease. Many different pathogenic fungi can cause these diseases (He, 2016). Leaf spots often lead to the destruction of over 50% of the infected leaves, resulting in serious economic losses (Lu et al., 2019; Qin et al., 2021; Hao et al., 2022; Yu et al., 2022). So far, we have identified 28 pathogenic fungi (excluding anthracnose and soft rot) that can cause *C. oleifera* leaf spot (Table 1). *Pestalotiopsis* spp and *Neopestalotiopsis* spp are the primary pathogenic fungi that cause *C. oleifera* leaf spot (Figures 1L, M). It is noteworthy that *Neopestalotiopsis* spp is a novel genus isolated from *Pestalotiopsis* spp, distinguished by analyses of systematics, sporulation structure, and conidial characteristics (Maharachchikumbura et al., 2014). *Pestalotiopsis* spp is a common pathogenic fungus, endophyte, and saprophyte. It is interesting to note that certain endophytic *Pestalotiopsis* spp can transform into a pathogenic phenotype, which can cause harm to the plant under specific conditions (Song, 2016). Conversely, when acting as an endophyte, it not only remains harmless but also produces a diverse array of compounds that assist the plant in resisting other pathogens (Rehman et al., 2022). Similar to other pathogenic fungi, the conidia of *Pestalotiopsis* spp have a crucial role in their pathogenicity, and any reduction in conidial numbers or functional deficiencies can significantly impact their ability to cause disease (Zhou et al., 2021; Yan et al., 2023). The pathogenic fungus overwinters in the form of mycelia or conidia in diseased leaf tissue and explodes in warm and humid conditions the following year, commonly in June and September, respectively (Liu et al., 2019; Lu et al., 2019). Some pathogenic fungi are more conducive to infestation during low temperatures and rainy weather (Kirk et al., 2008).

## 3 Geographical distribution of major fungal diseases of *C. oleifera*

Currently, there are 15 provinces (autonomous regions and municipalities directly under the central government) suitable for the cultivation of *C. oleifera* (National Forestry and Grassland Administration, 2023), primarily covering tropical monsoon, subtropical monsoon, and temperate monsoon climatic regions (Liu, 2019), all of which are characteristics by high temperature and rainy summer. It is highly conducive to the growth and spread of many pathogenic fungi. At present, *C. oleifera* anthracnose is currently the most serious disease, covering almost all the suitable areas for *C. oleifera* (Figure 1N). This is followed by soft rot, coal stain leaf gall, leaf spot, and root rot, which are also widespread (Figures 1O–S). There are only sporadic reports of white rot, scab disease, and algal spot (Figure 1T). *C. oleifera* leaf spot is difficult to distinguish because of the many different pathogenic fungi and the disease is likely to be confused with other foliar diseases. The actual extent of the real occurrence might be much larger than the areas depicted on the map, and we speculate that similar to anthracnose, it is present in all crucial production regions of *C. oleifera* in China. Due to global warming, the average temperature has increased, providing more opportunities for pathogens to thrive in the future (Roos et al., 2011). This means that more *C. oleifera* producing areas in China will be affected by more diseases in the future. *C. oleifera* plantation area in China is rapidly expanding, which also poses unprecedented challenges for disease management. According to the present statistical results, there are no significant geographical variations in the distribution of pathogenic fungi. Moreover, multiple pathogenic fungi can often be identified in *C. oleifera* stands, leading to the concurrence of several simultaneous diseases.

## 4 Management of fungal diseases in *Camellia oleifera*

Currently, the demand for tea oil and the associated quality requirements are on the rise in the international market, and there is also growing concern over domestic food safety. The development of the *C. oleifera* industry needs to maintain the excellent quality of tea oil while addressing the need to increase production significantly. To ensure the long-term health of *C. oleifera* forests, guarantee the high quality of tea oil, and prevent environmental pollution. It is necessary to establish a pollution-free management technology system that is founded on ecological management technology. Taking into account the entire ecosystem of the *C. oleifera* forest, we should establish an accurate monitoring and forecasting system, select and breed disease-resistant varieties, develop biological control techniques, and utilize chemical pesticides rationally to carry out integrated management. Both to management diseases in *C. oleifera* below the permissible economic threshold and to ensure the high and stable production of pollution-free tea oil. Currently, the management of *C. oleifera* diseases is primarily conducted through traditional chemical methods. The use of broad-spectrum fungicides increases both the cost of production and the risk of disease-causing fungal resistance. Although there are numerous methods for the prevention and control of plant diseases, each one possesses advantages, disadvantages, and limitations. Practice has demonstrated that no single control measure for any disease can entirely and efficiently address the issue of damage. Only by adapting measures to local conditions and implementing integrated disease management (IDM) can we achieve the best control effect by leveraging the strengths of various measures and addressing their shortcomings. However, the development of new methods and technologies for managing *C. oleifera* fungal disease is seriously lagging, which is not conducive to the sustainable development of the industry. In the context of *C. oleifera* fungal disease patterns and the pathogenic mechanisms of the causal fungi, we will discuss several potential management strategies.

### 4.1 Selection and breeding of disease-resistant varieties

Cultivating varieties that are resistant to diseases is considered the best way to manage plant diseases in the long term (de Resende et al., 2021). Crossbreeding and systematic selection are frequently employed in the selection of new *C. oleifera* varieties, while traditional selection methods, which are labor-intensive, time-consuming, and highly dependent on environmental conditions, have demonstrated limitations in the development of broad-spectrum disease-resistant varieties. Indeed, newly improved varieties are often unable to adapt to the frequent emergence of new virulent microspecies of pathogens. With the development of biotechnology and molecular biology, the use of molecular breeding can overcome the limitations imposed by traditional breeding. The technique of molecular marker-assisted selection (MAS) has found extensive application in variety selection. Molecular markers can improve the efficiency and precision of genetic analyses, particularly in cases where multiple resistance genes are present in a single variety (Paterson et al., 1991). The disease resistance (R) gene is characteristics by specific evolution, enables plants to recognize pathogen-specific Avr genes, initiate signal transduction to activate defense capabilities, and naturally become an ideal target. The causal fungi that cause *C. oleifera* disease typically complete the infestation through mechanical invasion, toxin production, or induction of cell death. However, R genes can prevent the invasion of pathogenic fungi by regulating the strength of structures such as the plant cell wall and callose in a relative manner (Bacete et al., 2020); Altering the target or direct detoxification of toxins secreted by pathogenic fungi (Wang et al., 2021); Scavenging excessively accumulated ROS while inhibiting cell death (Navarrete et al., 2022). R genes improve disease resistance in plants in various ways. Moreover, the proteins encoded by disease-resistance genes have much in common, which opens up the possibility of selecting broad-spectrum disease-resistant varieties. The mining of R genes within the *C. oleifera* genome is bound to significantly assist in the identification of *C. oleifera* varieties that possess broad-spectrum disease resistance. Naturally, the publication of genome-wide data for *C. oleifera* will make this task much easier (Lin et al., 2022). In addition to mining disease-resistance genes, another future task that needs to be completed is the construction of a mature *C. oleifera* tissue culture system. This will significantly shorten the selection cycle and minimize the impact on the environment. It has been reported that treating healing tissues with pathogenic fungal toxins can also induce plants to develop corresponding resistance under selection pressure (Hui et al., 2003). Furthermore, another method to obtain precisely resistant varieties is by utilizing tissue culture technology and gene editing technology to introduce genes encoding antimicrobial proteins into *C. oleifera*, resulting in the construction of disease-resistant transgenic plants. However, some legal, ethical, and experimental issues continue to hinder the commercial utilization of transgenic plants (Fritsche et al., 2018). However, few key disease resistance genes have been identified in *C. oleifera*, and there is a lack of a mature genetic transformation system. Currently, precise cultivation of plants with targeted resistance poses a challenge.

### 4.2 Establishing an accurate monitoring and forecasting system

Fungal diseases of *C. oleifera* can be said to be an important reason for the loss of *C. oleifera* yield. Therefore, accurate modeling and prediction of such diseases can facilitate advance understanding of the time and severity of disease outbreaks. The majority of pathogenic fungi, that cause *C. oleifera* diseases, are disseminated through the medium of spores. Pathogenic fungi produce a significant quantity of spores on affected tissues, including diseased branches, leaves, and fruits, which can then spread via air currents or animals or human activities, frequently leading to widespread disease outbreaks. As a result, the presence and variety of spores in the air are strongly linked to the incidence of diseases (Volz, 1997; Suffert et al., 2011; Mukherjee et al., 2021). The spore capture technology can collect and detect spores of pathogenic fungi in the air of a given area, and simultaneously integrate them with the phenological phase of *C. oleifera* and the corresponding meteorological factors. It can accurately predict and analyze the period and degree of occurrence of disease, as well as their spatial and temporal variations, in *C. oleifera* forest regions (Redondo et al., 2020; Galagedara et al., 2021; Miller et al., 2022). Initially, spore trapping techniques were frequently employed in conjunction with microscopic examination to identify and quantify spores, but the method entails a high degree of detection error, such that even seasoned professionals cannot ensure a high degree of accuracy. Moreover, the form and size of the spores of certain pathogenic fungi may vary over time. The qPCR technique is deemed to be the most dependable technique for measuring the relative levels of genes in various samples simultaneously, thanks to its high efficiency and sensitivity (Galli et al., 2015; Machado et al., 2015). Several studies have successfully combined spore-trapping techniques with qPCR to analyze the environmental spore densities of various plant pathogens (Dvořák et al., 2017; Quesada et al., 2018). It is noteworthy that a diverse array of fungal spores coexist in the air, encompassing non-pathogenic spores, and in addition, spore densities are lower in the air. Therefore, the specificity and sensitivity of qPCR protocols for the detection and quantification of *C. oleifera* pathogenic fungi spores are critical. The fungal ITS sequence is a suitable choice for the specificity requirement as it experiences minimal natural selection pressure during evolution and can accommodate greater variation. To achieve the best possible detection, the sensitivity can be optimized by optimizing primer sequences, DNA extraction techniques, and other factors. At present, there have been studies on high-precision detection technology for some *C. oleifera* diseases (Song, 2012), which provides support for improving the monitoring and prediction system for *C. oleifera* diseases. As a soil-borne disease, *C. oleifera* root rot is caused by a fungus that does not rely on conidia as its primary mode of transmission, making it unpredictable through the methods described above. Root rot is a so insidious disease, causing symptoms to go unnoticed in its early stages, and once the typical symptoms surface, they are often irreversible. Although the initial symptoms of root rot are not obvious, subtle changes in tissue metabolic pathways occur when plant tissues are attacked by the causal fungus. The NIR broad-spectrum method can rapidly identify this nuanced difference to diagnose the health status of the plant, making it effective for the early detection of plant root rot (Calamita et al., 2021). However, little research has been conducted on the differential changes in tissue metabolic pathways following infection by pathogenic fungi in *C. oleifera*, hindering the promotion and application of this technology in monitoring fungal diseases in this plant.

### 4.3 Use of biological control technology

Biological control is one of the practices of integrated plant disease management that uses biological agents to suppress the growth of pathogens, thereby preventing the development of plant disease (O’Brien, 2017; Kohl et al., 2019). These include the utilization of microbial antagonists like bacteria, fungi, viruses, or nematodes for disease suppression (Heydari and Pessarakli, 2010). Multiple studies have indicated that microorganisms isolated from the surface or inner part of plant tissues, as well as inter-root soil, can inhibit the growth of pathogenic bacteria, compete for colonization, or improve plant resistance (Dong et al., 2022; Kong et al., 2022; Sun Z. Z. et al., 2022; Zhou et al., 2022). Against *Colletotrichum* spp, a multitude of antagonistic microorganisms secrete extracellular enzymes, antibiotics, or volatile compounds that trigger mycelial disintegration, shrinkage, or abnormal conidial development of the pathogenic fungus, thus preventing its infestation (Bonatelli et al., 2016; Won et al., 2019). Furthermore, some antagonistic microorganisms can prevent disease by disrupting the cell wall structure of the parasitic mycelia (Alvindia, 2018). When selecting antagonistic microorganisms, it is important to consider the ability to colonize as well. The working principle of such antagonistic microorganisms is rapid dominance in plant tissues, access to more nutrients, and the simultaneous synthesis of more inhibitory substances. Moreover, possessing greater athleticism and the capability to ability to adapt to harsh environmental conditions is a necessary prerequisite for achieving success (Carvajal et al., 2002; Capdevila et al., 2004). Improving the plant’s resistance can better handle diverse disease-causing fungi. Undoubtedly, using beneficial microorganisms to indirectly improve the resistance of *C. oleifera* is an effective method in the practical setting of lacking highly resistant *C. oleifera* varieties. *Trichoderma* fungi and *Bacillus* bacteria are often applied for biological control. They can enhance plant growth by improving the soil environment or producing a range of secondary metabolites to stimulate the plant’s defense mechanism, thereby improving the plant’s resistance to diseases (Perelló and Bello, 2011; Miljakovic et al., 2020; Asad, 2022). In addition, the application of fungus-feeding nematodes has been found to effectively decrease the progression of soil-borne diseases (Hasna et al., 2008), and mycoviruses to control the incidence of sclerotinia blight (Jiang et al., 2013). The control techniques presented hold immense potential for managing fungal diseases of *C. oleifera*. The key to the effectiveness of microbial antagonists lies in obtaining high-quality microbial that have strong environmental adaptability and athletics, while maintaining good antibacterial activity. This results in an extremely high cost for the preparation and development of microbial agents. At the same time, the use of pharmaceuticals necessitates professional training. Therefore, high human and economic costs are one of the key constraints to the diffusion of microbial antagonists. In addition to microbial antagonists, plant-derived fungicides are also highly sought after by people. Many phytochemicals have the potential to suppress the progression of plant fungal diseases (Cho et al., 2007; Gahukar, 2018). Plant-derived fungicides have the characteristics of low toxicity to humans and can be rapidly degraded into non-toxic substances under natural conditions. The study discovered that aldehydes, phenols, alkaloids, and other substances derived from plants can impact the development of numerous plant pathogenic fungi, including *Fusarium* spp, *Colletotrichum* spp, and *Sclerotinia* spp (Cho et al., 2007). For example, cinnamaldehyde can inhibit the synthesis of chitin in fungal cell walls and inhibit the infection activity of *F. solani* (Wang J. et al., 2022); Berberine has the ability to suppress the growth of various pathogenic fungi, including *C. capsici* and *Pyricularia oryzae* Cav. by inhibiting the growth of fungal hyphae and the germination of spores (Singh et al., 2001; Kokkrua et al., 2019). Despite the fact that botanical fungicides have been used in production, their development has been hindered by factors such as unstable properties, susceptibility to degradation, and slow efficacy.

### 4.4 Reasonable use of chemical pesticides

In production practice, Fungicides such as carbendazim, Bordeaux mixture and thiophanate methyl are frequently employed to manage fungal diseases of *C. oleifera*. The indiscriminate use of these chemicals poses risks, including chemical residue transfer (Li Y. H. et al., 2017; Zhou et al., 2018) and severe plant toxicity (Gaikwad and Nimbalkar, 2005). They remain toxic to animals, animal embryos, and even humans (Fimognari et al., 1999; Ellis et al., 2010; Baurand et al., 2016). Apart from the safety issue, drug resistance is also a problem that cannot be disregarded. One of the key factors in the emergence of resistance is the reduction in protein affinity that is targeted by fungicides, resulting from a single target of drug action and a single gene mutation (e.g., carbendazim, thiophanate-methyl). Current chemical management methods, often in the disease-prone period of centralized application, and with a variety of agents used in combination, not only improve the efficiency of the use of chemical pesticides but also reduce the risk of drug resistance (Mueller et al., 2004). Fungicide resistance management is essential to slowing the emergence and spread of resistance for as long as possible while maintaining the necessary level of disease management. Developing a scientific pesticide application plan for the daily management of *C. oleifera* disease to reduce various risks is urgently needed in this regard. During the development of new chemical agents, the captivation of plant resistance using plant immune inducers can effectively achieve disease resistance (Caffi and Rossi, 2018). Immune inducers are mostly plant hormones or active small molecules produced during the interaction between pathogens and their hosts (Bektas and Eulgem, 2014), which do not induce resistance in disease-causing fungi as they do not significantly inhibit the pathogenic fungi themselves. They are also environmentally friendly with low toxicity to humans and animals, and can be used at low concentrations with a wide range of effects. The comprehensive and flexible application of plant immune inducers will facilitate the discovery of potential solutions to challenges related to food safety and agrochemicals (Guo et al., 2021). As a “vaccine” for plants, the development of drugs for immune inducers requires the search for corresponding “antigens,” which cannot be separated from the study of pathogenic mechanisms of pathogenic fungi. At present, the research on the pathogenic mechanism of pathogenic fungi in *C. oleifera* is still in its infancy, and the development of corresponding immune inducers is still very difficult.

## 5 Conclusion and outlook

**FIGURE 2:**
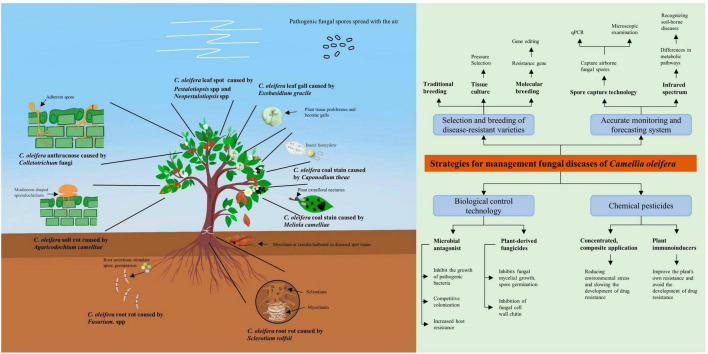
Schematic diagram of pathogenic bacteria infesting *Camellia oleifera* and integrated disease management strategies. This figure serves as an image summary outlining the basic elements of the literature review.

This review provides a more detailed understanding of *C. oleifera* diseases by describing the species, geographic distribution, and pathogenesis of the major fungal diseases (Figure 2). Among the numerous fungal diseases of *C. oleifera*, anthracnose caused by *Colletotrichum* spp has the widest distribution range. Moreover, *Colletotrichum* spp possesses numerous hosts and exhibits robust destructive power, often leading to cross-infection of different plants in the same forest (Eaton et al., 2021; Sharma et al., 2022). Therefore, *Camellia* anthracnose is the key object of prevention in daily production. The causal fungi responsible for fungal diseases of *C. oleifera* are diverse, with multiple diseases co-occurring, but there is no evidence of significant geographic variation in them. Therefore, the management of *C. oleifera* fungal diseases in all regions needs to maintain a high level of attention to multiple diseases at the same time. A comprehensive examination of the pathogenesis of each disease can facilitate the development of more effective targeted prevention and management strategies. As an example, *C. oleifera* root rot, as a soil-borne disease, is greatly limited in its ability to spread by the soil environment, with the causal fungus tending to only attack poorly healthy and young *C. oleifera* plants. Therefore, by implementing effective fertilizer management strategies to maintain seedlings healthy, along with regular disinfecting the of soil in the seedbed, *C. oleifera* root rot can be effectively management.

The management of fungal diseases in *C. oleifera* needs to combine a variety of measures. A single chemical management measure is often ineffective in suppressing the disease and plants are subjected to combined attacks by multiple pathogenic fungi (Yimer et al., 2018). The traditional methods of prevention and management have limitations. For example, an integrated management strategy that prioritizes selects of resistant varieties and combines them with rational chemical methods and cultivation measures can be more effective in minimizing losses caused by plant spotted wilt diseases (Culbreath et al., 2003). However, the present strategies for managing *C. oleifera* fungal disease remains relatively homogenous, and the advancement of new management strategies is inseparable from fundamental research on *C. oleifera* and the fungi responsible for causing the disease. Recent research should therefore focus on, but not be limited to, the following two areas. Firstly, the selection of resistant varieties is the best option to maximize the benefits of production. Not only is it environmentally friendly, but it’s also long-lasting. Therefore, it is worth identifying disease-resistance genes in *C. oleifera*. Secondly, a comprehensive understanding of the epidemiology and pathogenesis of various diseases, along with targeted early administration of drugs and the use of compound agents, would significantly reduce production costs and environmental hazards. This would also facilitate the establishment of accurate monitoring and prediction systems. Similarly, developing “specific drugs” for different diseases also requires a sufficient understanding of the pathogenic mechanisms of fungi. In addition, an in-depth understanding of the interactions between *C. oleifera* and pathogenic fungi will facilitate the development of highly effective targeting agents. For example, the development of plant immune inducers requires understanding the immune response mechanisms of *C. oleifera* in response to different pathogenic fungi, targeted stimulation of key gene regulation, and can effectively improve the overall disease resistance of *C. oleifera* without the risk of drug resistance. Furthermore, continuously screening high-quality microbial resources with the potential to develop microbial antagonists, establishing a database of plant-derived fungicide resources, and enhancing and optimizing extraction processes are directions of research that demand ongoing attention.

## Author contributions

XC, GZ, and JL contributed to the conception of the manuscript. ZW, YH, AN, YX, and DZ contributed to the data analysis. All authors have read and agreed to the published version of the manuscript.
