# Supplementary material for: Research progress and management strategies of fungal diseases in Camellia oleifera
Source: Front Microbiol. 2023 Nov 23;14:1215024. doi: 10.3389/fmicb.2023.1215024 (PMC10702231; doi:10.3389/fmicb.2023.1215024)
Supplement: Supplementary file 1 [file Table_1.DOCX]

**Supplementary**

Cao, H.M., Wang, G.L., Chen, J., Li, X., Liang, Y.P., and Wei, H.Y. (2015). Investigation on the diseases and insect pests of tea-oil tree(Camellia oleifera)in Meiling mountainous region near Nanchang. *Plant Protection* 41(01)**,** 166-170.

Cao, Z.H., Su, Q.L., and Zhang, X. (2011). Occurrence and identification of oil tea diseases in Anhui. *Anhui Forestry Science and Technology* 37(01)**,** 55-58.

Chaling Academy of Forestry (1976). A trial of garlic liquid for the control of anthracnose on oil tea. *Hunan Forestry Science & Technology* (04)**,** 26-27.

Changde Academy of Forestry (1973). Talking about technical measures for high and stable yield of oil tea. *Hunan Forestry Science & Technology* (06)**,** 12-13.

Chen, J. (2016). *Investigation on the occurrence of main disease and insect pests in Camellia oleifera forest in central region of Jiangxi province.* [master's thesis] Nanchang(Jiangxi): Jiangxi Agricultural University.

Chen, J.X., Wei, Y.Q., Liu, L., Zhang, D.H., Ma, H.C., and Wu, J.R. (2022). Identification of Camellia oleifera anthrax pathogens in Yunnan province and screening of antagonistic bacteria. *Journal of South China Agricultural University* 43(05)**,** 43-53.

Chen, S.C., Tian, Z.J., and Chen, D.C. (1964). Morphological characteristics, frequency of occurrence, pathogenicity and conditions of production of sexual generations of the anthracnose fungus of oilseed tea. *Acta Phytopathologica Sinica* (01)**,** 45-52. doi: 10.13926/j.cnki.apps.1964.01.005.

Chen, Y.S., and Kang, X.Z. (2005). Three major diseases of oil tea and their control measures. *Anhui Forestry* (03)**,** 44.

Chen, Z.L., Yuan, Y., Hu, M.H., Huang, X.Y., Li, W.H., Wang, Y., et al. (2018). An investigation of the pest of oil-tea(Camellia oleifera) in Pingjiang county. *Hunan Forestry Science & Technology* 45(05)**,** 90-95.

Deng, X.Z., Huang, L.G., and Deng, Y.W. (2011). Study on the effect of five agents on the control of anthracnose in oil tea. *Journal of Anhui Agricultural Sciences* 30(16)**,** 9653-9654. doi: 10.13989/j.cnki.0517-6611.2011.16.166.

Deng, Y., Li, D.W., Jiang, X.J., and Wu, Y.J. (2013). Species investigation on the diseases and insect pests of Camellia oleifera and their occurrence characteristics in Guangxi. *Guangxi Forestry Science* 42(04)**,** 339-346. doi: 10.19692/j.cnki.gfs.2013.04.010.

Fang, L.X. (1985). Analysis of the benefits of yield increase after refurbishment of old-growth oil tea forests. *Non-wood Forest Research* (02)**,** 63-65.

Fujian Forestry College, and Forest Protection Teaching and Research Group (1966). A summary of experience in squatting to control anthracnose in oil tea. *Scientia Silvae Sinicae* (03)**,** 9-14.

Guangxi Academy of Forestry (1965). Control of anthracnose in oil tea. *Forest Science and Technology* (18)**,** 9-10. doi: 10.13456/j.cnki.lykt.1965.18.001.

H, Y., Wei, W., Wu, B., Shu, M.S., Wang, D.X., and Jiang, Z.P. (2014). Isolation and primary identification of th pathogen of anthracnose in Camellia oleifera. *Acta Agriculturae Universitatis Jiangxiensis* 36(02)**,** 314-318. doi: 10.13836/j.jjau.2014051.

He, X.H. (2009). Common oil tea pest control techniques. *Hunan Forestry* 618(02)**,** 28-29.

He, X.Y., and Cai, S.P. (2013). Present situation and control measures of disease and insect ppest in Camellia oleifera forest in Fujian province. *Journal of Fujian Forestry Science and Technology* 40(04)**,** 181-184+188.

He, Z., Zhou, G., Li, M., Wu, Y.P., Xia, Y.G., Yan, X.W., et al. (2013). Pest and disease situation of oil tea in Hunan Province and its characteristics. *Insect Studies in Central China* 9(00)**,** 163-169.

Huang, C.W. (2010). Main pests and diseases and integrated control measures of Camellia oleifera in Dehua County. *Subtropical Agriculture Research* 6(02)**,** 106-110. doi: 10.13321/j.cnki.subtrop.agric.res.2010.02.014.

Huang, L.L. (2012). Current status of oil tea development in Ningguo City and control measures of soft rot disease. *Anhui Agricultural Science Bulletin* 18(08)**,** 108-109.

Huang, X.H. (2000). Trials on the efficacy of three agents including chlorothalonil for the control of anthracnose of oil tea. *Jiangxi Forestry Technology* (02)**,** 18-19. doi: 10.16259/j.cnki.36-1342/s.2000.02.011.

Ji, Z.P., and Guo, X.S. (1992). Characteristics of anthracnose in oil tea and its control. *Shaanxi Forest Science and Technology* (04)**,** 70-72.

Jia, D.S., Miao, J.H., Chen, F., Miao, M., H, and Liao, Y.J. (2017). Occurrence and control of Camellia oleifera gall disease induced by exobasidium gracile in highland areas. *Journal of West China Forestry Science* 46(05)**,** 29-34+51. doi: 10.16473/j.cnki.xblykx1972.2017.05.006.

Jiang, C.Y., and Shi, S.L. (2013). Investigation and control of major pest and disease species of oil tea in Jinping County. *Agricultural Technology Service* 30(08)**,** 832-833.

Jin, J. (2011). Control of three major diseases of oil tea. *Forestry of China* 738(17)**,** 27.

Li, H. (2013). Common diseases and control techniques of oil tea in Fuan. *Forest By-Product and Speciality in China* 124(03)**,** 68-69. doi: 10.13268/j.cnki.fbsic.2013.03.020.

Li, X.M. (1984). Oil tea anthracnose control trials. *Hunan Forestry Science & Technology* (01)**,** 31-32.

Li, Y., Li, H., Zhou, G.Y., Jiang, Y.X., and Liu, J.A. (2016). Virulence and biological characteristics of three pathogens causing oil tea anthracnose in Hainan province. *Chinese Journal of Tropical Crops* 37(10)**,** 1956-1961.

Li, Y., Liu, W.L., Jian, H.Y., Guo, S.L., Li, Z.Q., and Nie, C.P. (2012). Climatic patterns, effects and control of oil tea pests and diseases in Yuanzhou District. 271(09)**,** 102.

Li, Z.S. (2012). Control techniques for major pests and diseases of oil tea. *Forest By-Product and Speciality in China* 121(06)**,** 64-65. doi: 10.13268/j.cnki.fbsic.2012.06.010.

Liang, Q. (2009). Oil tea anthracnose and control. *Farmers' Friend (Theory Edition)* 278(08)**,** 59-60.

Liang, Z.C., Cen, B.Z., and Dai, P.C. (1964). Sexual generations, overwintering patterns and invasion pathways of the anthracnose fungus of oilseed tea. *Journal of Plant Protection* (03)**,** 316-317.

Lin, M.S. (2015). Investigation and prevention of disease on Camellia oleifera in Minhou county. *Journal of Fujian Forestry Science and Technology* 42(02)**,** 150-153. doi: 10.13428/j.cnki.fjlk.2015.02.033.

Liu, J.A., Pan, H.P., Wu, N., and Li, H. (2010). Spatial distribution pattern of major diseases in Camellia oleifera. *Forest Pest and Disease* 29(05)**,** 7-10.

Liu, S.B. (2011). *A preliminary study of root rot pathofen on Camellia.* [master's thesis] Wuhan(Hubei): Huazhong Agricultural University.

Liu, W., Wang, Y.X., Chen, J.Y., Zheng, L., and Huang, J.B. (2011). Preliminary study on the identification of anthracnose pathogen of oil tea and the overwintering of the pathogen. *Proceedings of the 2011 Annual Academic Conference of the Chinese Society of Plant Pathology*.

Lu, D.S., Huang, X.H., and B, D. (2012). Survey and identification of Camellia oleifera diseases in Xinyang area. *Journal of Northeast Forestry University* 40(05)**,** 83-85+111. doi: 10.13759/j.cnki.dlxb.2012.05.013.

Luo, H., Liang, Y., Xiao, Q.L., Liao, Z., and Liu, S.H. (2011). Trial of Fructis to control anthracnose in grafted oil tea seedlings. *Forest Pest and Disease* 30(03)**,** 46.

Luo, L.N., Han, S.Q., Wei, H.T., Wang, G.J., Zhang, K.Y., Li, Z.F., et al. (2020). Survey and Integrated Control of Major Pests and Diseases of Oil Tea in Wangmu County. *Bulletin of Agricultural Science and Technology* 588(12)**,** 267-269.

Miao, J.H., Jia, D.S., Chen, F., Miao, M., H, Liao, Y.J., Guo, X.C., et al. (2016). A preliminary study on oil tea fruit drop in high mountains. *Forest Science and Technology* 520(04)**,** 23-26. doi: 10.13456/j.cnki.lykt.2016.04.006.

Ni, B.L. (1979). Investigation of the causes of anthracnose in oil tea in Gaozhou. *Forestry and Environmental Science* (03)**,** 32-34.

Qin, S.Z., Deng, J., and Wang, J.W. (2020). Identification of the pathogen of the leaf spot disease Lasiodiplodia theobromae of oil tea in Guizhou. *Shanxi Agricultural Economy* 265(01)**,** 90-91. doi: 10.16675/j.cnki.cn14-1065/f.2020.01.050.

Qiu, J.S., Yu, J.Y., Wu, Y.K., Zhu, X.E., Wang, J., and Xu, J. (2011). Preliminary report on the Ecxxobasidium gracile(Shirai)Syd. of Camellia oleifera in Guizhou province. *Guizhou Forestry Science and Technology* 39(01)**,** 19-22.

Rao, H.F., Ding, K.M., Rao, Y.P., Zhang, S.L., and Hong, H.L. (2013). Occurrence and control of major diseases of oil tea. *Plant Health and Medicine* 26(03)**,** 16-17+12. doi: 10.13718/j.cnki.zwys.2013.03.014.

Ruan, L.X., Wu, J.G., and Lin, X.J. (1998). Identification and control trials of soft rot of oil tea. *Forestry Technology Development* (01)**,** 37-39. doi: 10.13360/j.issn.1000-8101.1998.01.015.

Shaoguan Academy of Forestry (1974). Causes of low yields of oil tea and future management measures. *Forestry and Environmental Science* (03)**,** 20-22.

Shi, H.A., Fu, B.Z., Zhang, Z.L., Li, Y.G., and Wang, L.H. (2015). The study of two Camellia pathogens isolation, identification and biological characteristics. *Hubei Agricultural Sciences* 54(23)**,** 5908-5911. doi: 10.14088/j.cnki.issn0439-8114.2015.23.028.

Shi, S.D., and Cheng, Y.M. (2016). The current situation of anthracnose in oil tea in western Anhui and its control measures. *Anhui Science & Technology* 333(11)**,** 39-40.

Shu, J.P., Teng, Y., Liu, J., Zhang, Y.B., and Wang, H.J. (2013). Preliminary analysis on the causes of pre-harvest fruit drop in Camellia oleifera. *China Plant Protection* 33(01)**,** 9-14.

Tan, S.S. (1959). Preliminary Survey of Oil Tea Diseases. (33)**,** 7-8. doi: 10.13456/j.cnki.lykt.1959.33.005.

Tang, Y.L., Zhou, G.Y., Li, H., Zhong, W.B., Gong, H.E., and Wang, L.Y. (2015). Identification of a new anthracnose of Camellia oleifera based on multiple-gen phylogeny. *Chinese Journal of Tropical Crops* 36(05)**,** 972-977.

Tian, Y., Zhang, G.H., Zhang, L.C., Qin, Y., Chen, G.F., Lou, L.H., et al. (2021). Investigation on the main diseases and insect pests of Camellia oleifera in Chongqing. *Journal of Anhui Agricultural Sciences* 40(20)**,** 163-165+180.

Wang, C.T. (2022). Occurrence status and pollution-free control methods of insect pests and diseases of Camellia oleifera in Chengmai. *Tropical Forestry* 47(04)**,** 66-69.

Wang, J., Chen, S.H., Huang, Y.F., and Sun, S. (2006). Induced resistance to anthracnose of Camelia oleifera by salicylic acid. *Forest Research* (05)**,** 629-632.

Wang, W.W. (2021). The main diseases and insect pests of Camellia oleifera in She county. *Forest By-Product and Speciality in China* 172(03)**,** 5-58+60. doi: 10.13268/j.cnki.fbsic.2021.03.022.

Wang, Z.C., Zhang, T., and Zhu, X.T. (2022). Ecological cultivation technology of Camellia oleifera in the Ta-pieh mountains of western Anhui. *Horticulture & Seed* 42(12)**,** 77-78+81. doi: 10.16530/j.cnki.cn21-1574/s.2022.12.029.

Wei, M., Lu, L., Li, C.Q., Fu, B.Z., Li, G.Y., and Wang, L.H. (2016). Identification of antagonistic fungus to Camellia diseases and study of biological characteristics and antagonism effect. *Journal of Henan Agricultural Sciences* 45(08)**,** 74-80. doi: 10.15933/j.cnki.1004-3268.2016.08.014.

Wen, Y.X., Liu, J.A., Liu, X.P., and Zhou, G.Y. (2016). Effect of different interplanting patterns on occurrence of diseases and insect pests in Camellia oleifera. *Non-wood Forest Research* 34(01)**,** 129-134. doi: 10.14067/j.cnki.1003-8981.2016.01.021.

Wong, Y.X., Zhang, C.Q., Zhao, H.M., Xu, N.H., and Yang, W.Q. (1982). Research on anthracnose of oil tea spring tips. *Scientia Silvae Sinicae* (03)**,** 289-295.

Wu, G.J., Lin, X.J., and Yin, H.Q. (1993). Studies on the resistance of different species, varieties and types of oil tea to anthracnose. *Non-wood Forest Research* (S1)**,** 318-322.

Wu, J., and Huang, J.J. (2015). Occurrence of common pests and diseases of oil tea in Yuping County and control measures. *Modern Agriculture* 472(10)**,** 39. doi: 10.14070/j.cnki.15-1098.2015.10.030.

Wu, T. (2008). Major pests and diseases of oil tea in Jiangxi and their control techniques. *Modern Agricultural Science and Technology* 473(03)**,** 92-93.

Wuyi County Forestry (1977). Preliminary summary of integrated control of anthracnose on oil tea. *Journal of Zhejiang Forestry Science and Technology* (02)**,** 13-15.

Xiao, B., and Xiao, L. (2018). Present situation and countermeasures against Camellia oleifera pests and diseases in Dayu county, Jiangxi province *Biological Disaster Science* 41(03)**,** 213-217.

Xie, Q.M., Jiang, J., and Huang, J.H. (1989). Physiological and biochemical effects of anthrax toxins on different resistant oilseed tea species. *Journal of Fujian Forestry College* (02)**,** 101-107.

Xin, S.S., Qi, G.F., Zhu, F.Y., Lu, J.Z., Li, Q.Q., Wang, S.Y., et al. (2011). Optimization of fermentation condition for Bacillus amyloliquefaciens WH1 and its biological control effect on Colletotrichum gloeosporioides. *Journal of Huazhong Agricultural University* 30(04)**,** 411-415. doi: 10.13300/j.cnki.hnlkxb.2011.04.007.

Yan, Q., Yang, W.X., Deng, Y., Tang, J., Deng, X.J., Qing, Q.Y., et al. (2013). Occurrence and control of the diseases and insect pests on oil-tea plants(Camellia oleifera Abel) in Guangxi. *Plant Protection* 39(02)**,** 170-173.

Yang, J. (2007). Occurrence and control of major pests and diseases of oil tea. *Guangdong Agricultural Sciences* 208(07)**,** 66-68. doi: 10.16768/j.issn.1004-874x.2007.07.023.

Yang, Q.X., and Luo, Y.S. (2010). Investigation and analysis of factors influencing the survival rate of oil tea plantations. *Modern Agricultural Science and Technology* 524(06)**,** 206-213.

Yongxing Academy of Forestry (1977). Soil pesticide control of anthracnose on oil tea. *Hunan Forestry Science & Technology* (03)**,** 22-23.

Yu, C.G., and Liu, Q. (2021). Control measures for major pests and diseases during the growing season of oil tea in Huanglong Forestry. *Agricultural Engineering Technology* 41(20)**,** 42-44. doi: 10.16815/j.cnki.11-5436/s.2021.20.025.

Yu, J.X., Nie, Y.A., Zhou, G., Li, M., Liao, Z.Q., Xia, Y.G., et al. (2014). Occurrence regularity of main disease of Camellia oleifera in Hunan. *Hunan Forestry Science & Technology* 41(01)**,** 94-97.

Yu, M.J. (2011). The incidence of anthracnose in oil tea and its control measures. *Anhui Agricultural Science Bulletin* 17(18)**,** 85-86.

Yu, Y.C. (2019). Occurrence and control of oil tea sooty mold in Susong area. *Journal of Agricultural Catastrophology* 9(02)**,** 17-18. doi: 10.19383/j.cnki.nyzhyj.2019.02.008.

Zhai, J.Z., and Huang, J. (2010). Pest and disease control of safflower oil tea introduced in Shanghai. 106(10)**,** 30. doi: 10.13456/j.cnki.lykt.2010.10.027.

Zhan, S.H. (2000). Chenglin oil tea yield demonstration trial. *Forest Science and Technology* (07)**,** 19-21. doi: 10.13456/j.cnki.lykt.2000.07.010.

Zhan, Z.R., Chen, X.H., Zhang, L.H., Zhang, D.Q., and Ye, C.Z. (2010). Major pests and diseases of oil tea in Youxi County and their control techniques. *Forest By-Product and Speciality in China* 104(01)**,** 40-43. doi: 10.13268/j.cnki.fbsic.2010.01.038.

Zhang, Y.H., Pu, F.J., Zhou, C.T., Zhang, L.Y., and Yan, H.X. (2017). Major pests and diseases of Xinyang oil tea and their occurrence. *Chinese Horticulture Abstracts* 33(04)**,** 97-99.

Zhangzhou Academy of Forestry (1973). A preliminary study on anthracnose of oil tea. *Forest Science and Technology* (03)**,** 15-16. doi: 10.13456/j.cnki.lykt.1973.07.009.

Zhao, D.Y., Qin, C.S., Jie, Y.Z., Xu, J.Z., and Yang, H. (2012). Investigation on diseases and pests species of Camellia oleifera and their occurrence dynamics in Guangdong. *Journal of Anhui Agricultural Sciences* 40(29)**,** 14267-14270. doi: 10.13989/j.cnki.0517-6611.2012.29.259.

Zhao, Z.X., Yan, W.R., Xiao, M., Xiao, D.B., and Lei, F. (2020). Molecular identification of pathogens causing root rot of Camellia oleifera in tropical. *Molecular Plant Breeding* 18(19)**,** 6433-6440. doi: 10.13271/j.mpb.018.006433.

Zhejiang Academy of Forestry (1962). A preliminary study on anthracnose of oil tea. (01)**,** 76-79.

Zhou, L.X. (2015). *Investigation and analysis of Camellia oleifera fruit drop reasons in Guangxi.* [master's thesis] Changsha(Hunan): Central South University of Forestry and Technology.

Zhou, X.Y. (2014). Characteristics of major pests and diseases of oil tea in Liancheng County and their control methods. *Modern Agricultural Science and Technology* 632(18)**,** 150-151.

Zhou, Y.T., Wang, F., Yin, J.B., Liu, L., Zhang, D.H., Hong, Y.D., et al. (2021). Screening and identification of Bacillus tequilensis 6157 as biological agent against Camellia oleifera anthracnose disease in Dehong prefecture. *Journal of West China Forestry Science* 50(04)**,** 131-138. doi: 131-138.DOI:10.16473/j.cnki.xblykx1972.2021.04.021.

Zhu, D.X., Zhou, G.Y., Xu, J.P., Liu, X.A., and Li, H. (2015a). Population genetic structure of Colletotrichum fructicola. *Mycosystema* 34(03)**,** 366-374. doi: 10.13346/j.mycosystema.140040.

Zhu, F., Zeng, T.W., Peng, W.W., and Deng, X.Y. (2018). The main pests and diseases of oil tea in northern Ganzhou and their control measures. *Modern Agricultural Science and Technology* 712(02)**,** 129-131.

Zhu, H., Qin, W.Q., Fu, D.Q., and Qi, Z.Q. (2015b). Identification and biological characteristics of the pathogen of oil tea anthracnose in Hainan. *Guangdong Agricultural Sciences* 42(16)**,** 55-59+54. doi: 10.16768/j.issn.1004-874x.2015.16.014.

(Tan, 1959; Zhejiang Academy of Forestry, 1962; Chen et al., 1964; Liang et al., 1964; Guangxi Academy of Forestry, 1965; Fujian Forestry College and Forest Protection Teaching and Research Group, 1966; Changde Academy of Forestry, 1973; Zhangzhou Academy of Forestry, 1973; Shaoguan Academy of Forestry, 1974; Chaling Academy of Forestry, 1976; Wuyi County Forestry, 1977; Yongxing Academy of Forestry, 1977; Ni, 1979; Wong et al., 1982; Li, 1984; Fang, 1985; Xie et al., 1989; Ji and Guo, 1992; Wu et al., 1993; Ruan et al., 1998; Huang, 2000; Zhan, 2000; Chen and Kang, 2005; Wang et al., 2006; Yang, 2007; Wu, 2008; He, 2009; Liang, 2009; Huang, 2010; Liu et al., 2010; Yang and Luo, 2010; Zhai and Huang, 2010; Zhan et al., 2010; Cao et al., 2011; Deng et al., 2011; Jin, 2011; Liu, 2011; Liu et al., 2011; Luo et al., 2011; Qiu et al., 2011; Xin et al., 2011; Yu, 2011; Huang, 2012; Li et al., 2012; Li, 2012; Lu et al., 2012; Zhao et al., 2012; Deng et al., 2013; He and Cai, 2013; He et al., 2013; Jiang and Shi, 2013; Li, 2013; Rao et al., 2013; Shu et al., 2013; Yan et al., 2013; H et al., 2014; Yu et al., 2014; Zhou, 2014; Cao et al., 2015; Lin, 2015; Shi et al., 2015; Tang et al., 2015; Wu and Huang, 2015; Zhou, 2015; Zhu et al., 2015a; Zhu et al., 2015b; Chen, 2016; Li et al., 2016; Miao et al., 2016; Shi and Cheng, 2016; Wei et al., 2016; Wen et al., 2016; Jia et al., 2017; Zhang et al., 2017; Chen et al., 2018; Xiao and Xiao, 2018; Zhu et al., 2018; Yu, 2019; Luo et al., 2020; Qin et al., 2020; Zhao et al., 2020; Tian et al., 2021; Wang, 2021; Yu and Liu, 2021; Zhou et al., 2021; Chen et al., 2022; Wang, 2022; Wang et al., 2022)
